# An Integrated Analysis of miRNA, lncRNA, and mRNA Expression Profiles

**DOI:** 10.1155/2014/345605

**Published:** 2014-06-18

**Authors:** Li Guo, Yang Zhao, Sheng Yang, Hui Zhang, Feng Chen

**Affiliations:** Department of Epidemiology and Biostatistics, School of Public Health, Nanjing Medical University, Nanjing 211166, China

## Abstract

Increasing amounts of evidence indicate that noncoding RNAs (ncRNAs) have important roles in various biological processes. Here, miRNA, lncRNA, and mRNA expression profiles were analyzed in human HepG2 and L02 cells using high-throughput technologies. An integrative method was developed to identify possible functional relationships between different RNA molecules. The dominant deregulated miRNAs were prone to be downregulated in tumor cells, and the most abnormal mRNAs and lncRNAs were always upregulated. However, the genome-wide analysis of differentially expressed RNA species did not show significant bias between up- and downregulated populations. miRNA-mRNA interaction was performed based on their regulatory relationships, and miRNA-lncRNA and mRNA-lncRNA interactions were thoroughly surveyed and identified based on their locational distributions and sequence correlations. Aberrantly expressed miRNAs were further analyzed based on their multiple isomiRs. IsomiR repertoires and expression patterns were varied across miRNA loci. Several specific miRNA loci showed differences between tumor and normal cells, especially with respect to abnormally expressed miRNA species. These findings suggest that isomiR repertoires and expression patterns might contribute to tumorigenesis through different biological roles. Systematic and integrative analysis of different RNA molecules with potential cross-talk may make great contributions to the unveiling of the complex mechanisms underlying tumorigenesis.

## 1. Introduction

Large-scale, genome-wide analyses have indicated that much of the human genome is transcribed, yielding a great many nonexonic transcripts [1, 2]. These nonribosomal and nonmitochondrial RNAs, which are metaphorically considered ribosomal dark matter, are quite abundant in cells. The transcription profile of the entire genome at a specific space and time can be obtained using microarray and sequencing technologies [3]. Noncoding RNAs (ncRNAs), including microRNAs (miRNAs), and long noncoding RNAs (lncRNAs) can be obtained to attract considerable attention of researchers in many fields.

miRNAs, a class of small ncRNAs (*≈*22 nt), are highly important regulatory molecules, and they have seen a great deal of study [4, 5]. Posttranscriptional gene regulation via miRNA is crucial to the regulation of gene expression. These small, single-stranded RNAs negatively regulate gene expression through partial base-pairing with target messenger RNAs (mRNAs). This influences the process of mRNA degradation or repression of translation [4, 6]. They have multiple roles in various biological processes that affect basic cellular functions, including cell proliferation, differentiation, death, and tumorigenesis [7]. Abnormal expression of specific miRNAs has been characterized as a common feature of human diseases, especially for malignancies. In these cases, genes encoding miRNAs may act as oncogenes, oncomiRs, or tumor suppressors [7–9]. Widely concerned lncRNAs are normally longer than 200 nucleotides. Studies have shown them to be involved in a broad range of important cellular processes, including chromatin modification, RNA processing, and gene transcription and that they do so through interaction with DNA and proteins [10–14]. LncRNAs are characterized as complex, diverse ncRNAs. They are usually involved in exons and introns and have 5′ cap and some of the features of mRNAs [15]. The larger ncRNAs have been shown to regulate gene expression through various mechanisms, such as complementary binding to protein-coding transcripts in the form of cis-antisense lncRNAs [16–20]. They also modulate transcription factors by acting as coregulators [19, 21–25]. Dysregulation of ncRNAs contributes to many biological processes by interfering with gene expression. In recent years, this has become a hot research topic as core regulatory molecules.

Although the many biological roles of ncRNAs have drawn a great deal of concern, systematic and integrative analyses of many kinds of RNA molecules (including functional mRNAs) have been rare. An integrated, genome-wide analysis involving many different RNA molecule levels is necessary if the complex regulatory network and mechanisms underlying tumorigenesis are to be understood. We ever performed analyses of miRNA-mRNA and miRNA-miRNA interactions using miRNA and mRNA expression profiles [26], but it is not enough to further understand the potential relationships between different RNA molecules, especially involving the novel concerned lncRNAs. In the present study, the close relationships between ncRNAs and mRNAs were examined through simultaneously profiling of miRNA, lncRNA, and mRNA in HepG2 and L02 cells using high-throughput technologies. An integrative method of analysis was developed to detect and comprehensively analyze the relationships between RNA molecules, especially between abnormally expressed miRNA, lncRNA, and mRNA molecules in tumor cells. A systematic analysis of miRNAs was also performed at the isomiR level. The results of the present study will enrich the genome-wide analysis of different molecules with potential cross-talk and contribute to further systematic studies of tumorigenesis.

## 2. Materials and Methods

### 2.1. Cell Culture and RNA Isolation

HepG2 and L02 cells were obtained from the American Type Tissue Collection. These were maintained in DMEM containing 10% FBS, 100 U/mL benzylpenicillin and 100 U/mL streptomycin at 37°C in a humidified 95% air 5% CO_2_ incubator. Total RNAs were isolated using TRIzol reagent (Invitrogen) according to the manufacturer's protocol.

### 2.2. Small RNA Sequencing and Microarray Experiments

Total RNA from each sample was used to prepare the small RNA sequencing library to perform sequencing on a Genome Analyzer IIx, which was used in accordance with the manufacturer's instructions, and was prepared for microarray hybridization. The raw small RNA sequencing data can be available in the Sequence Read Archive (SRA) database (http://www.ncbi.nlm.nih.gov/sra, accession number SRA 1262121), and the mRNA and lncRNA microarray data can be available in the European Molecular Biology L aboratory-Euro-pean Bioinformatics Institute (EMBL-EBI) database (http://www.ebi.ac.uk/).

### 2.3. Data Analysis

The total raw miRNA sequencing reads were first filtered using a Solexa CHASTITY quality control filter. The remaining sequencing reads were deleted the 3′ adapter sequences, and tags shorter than 15 nt were discarded. Then, the reads were aligned to the known human miRNA precursors (pre-miRNAs) in the miRBase database (Release 18.0, http://www.mirbase.org/) using Novoalign software (v2.07.11, http://www.novocraft.com/) [27]. Only one mismatch was allowed. Reads with counts under 2 were discarded when miRNA expression was calculated. According to recent reports on multiple isomiRs from a given miRNA locus [28–34], isomiRs (including those isomiRs with 3′ nontemplate additional nucleotides) were also comprehensively surveyed. Sequences that matched the pre-miRNAs in the mature miRNA region ±4 nt (no more than 1 mismatch) were defined as isomiRs. The original sequence counts of miRNAs were normalized to RPM (reads per million), and miRNA expression analysis was performed based on these normalized data at the miRNA and isomiR level.

Images from microarray were analyzed with Agilent Feature Extraction software (version 10.7.3.1). Raw signal intensities of mRNAs and lncRNAs were normalized using the quantile method and the GeneSpring GX v12.0 software package (Agilent Technologies). After quantile normalization of the raw data, lncRNAs and mRNAs for which 2 out of 2 samples had flags in the present or marginal were chosen for further data analysis.

Fold change was calculated to assess expressed miRNA profiles that were differentially expressed between the two samples at miRNA and isomiR levels. Differentially expressed lncRNAs and mRNAs were also identified through fold change filtering. To obtain abnormal ncRNA/mRNA species and filter out rare species with lower expression levels, fold change values were assessed by adding an additional low number (10 units) based on normalized datasets. Hierarchical clustering was performed using Cluster bb3.0 and TreeView 1.60 programs (http://rana.lbl.gov/eisen/) [35, 36]. Experimentally validated target mRNAs of aberrantly expressed miRNAs were collected from the miRTarBase database [37]. For miRNAs with few or no validated target mRNAs, the putative target mRNAs were integrated using the prediction software programs Pictar, TargetScan, and miRanda programs [38]. The threshold values were simultaneously controlled (e.g., in TargetScan, the threshold of total context score was less −0.30). The collected target mRNAs were further screened based on abnormally expressed mRNA profiles. Then, pathway and GO analysis were used to determine the roles of these differentially expressed mRNAs. Using CapitalBio Molecule Annotation System V4.0 (MAS, http://bioinfo.capitalbio.com/mas3/), further functional enrichment analysis was performed. Functional interaction networks were constructed using Cytoscape v2.8.2 Platform [39].

### 2.4. Schema for Integrative Analysis of ncRNA-mRNA Data

**Figure 1:**
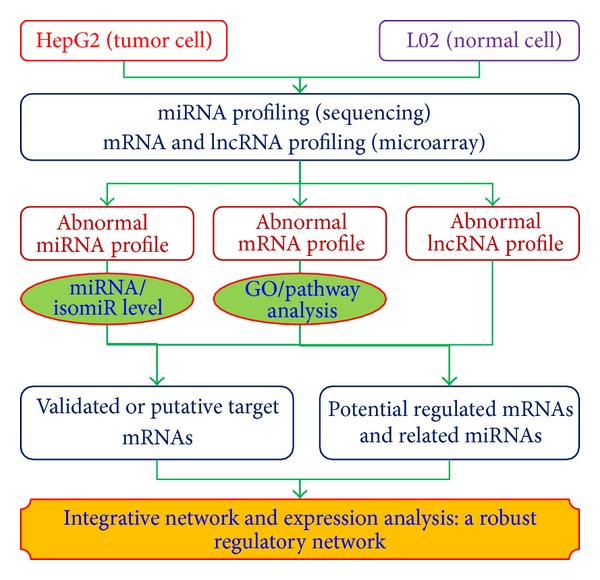
Integrative analysis of ncRNA-mRNA.

ncRNA-mRNA integrative analysis was performed according to Figure 1. The approach included three steps. First, profiles of aberrantly expressed miRNA, mRNA, and lncRNA in HepG2 cells were comprehensively surveyed using high-throughput datasets. A profile of aberrantly expressed isomiRs was obtained at the same time. Pathway and GO analyses were performed for abnormal mRNA and the target mRNAs of abnormal miRNAs. Second, systematic bioinformatic analysis was developed based on possible functional relationships between these molecules. miRNA and mRNA were analyzed in an integrated fashion based on experimentally validated or predicted target mRNAs and on their levels of enrichment. Possible internal relationships among lncRNA-mRNA and lncRNA-miRNA were identified based on their locational distributions and the relationships between their sequences. Finally, integrative regulatory network and expression analyses were performed at different molecular levels based on their possible levels of expression and functional relationships (Figure 1).

## 3. Results

### 3.1. Aberrantly Expressed miRNA and isomiR Profiles

As expected, 22 nt was the most common length (see Figure S1A and Figure S1B in Supplementary Material available online at http://dx.doi.org/10.1155/2014/345605). As found by Guo et al., consistent aberrantly expressed miRNAs were obtained based on the most abundant isomiR and all the isomiRs, respectively [34]. However, despite the consistency of the dysregulation pattern, the fold change (log 2) of some miRNAs was found to cover a wide range (miR-200b-3p: 8.56 and 5.45, miR-100-5p: −7.27 and −4.88) (Table 1). Half of the most dominant isomiR sequences had lengths that were different from those of canonical miRNA sequences (data not shown). Generally, the most dominant isomiR sequence may be longer or shorter than registered miRNA sequence through altering 5′ and 3′ ends, especially for the 3′ ends (Figure S1C). These abundantly and abnormally expressed miRNAs were always located on a few specific chromosomes, especially chromosome 9 (Figure S1D). The distribution bias was obvious, even though multicopy pre-miRNAs were also analyzed. Downregulated miRNAs were found to be more common than upregulated species, although the total numbers remained similar across the whole abnormal miRNA profiles.

**Figure 2:**
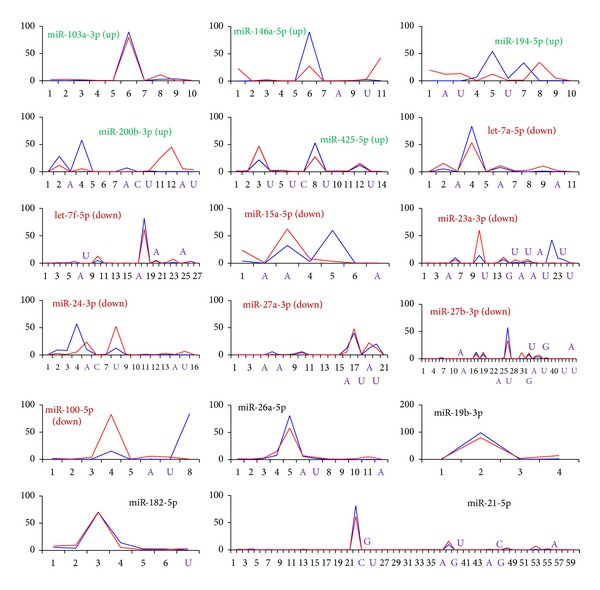
Various isomiR profiles. These miRNAs are the most abundantly up- (green) or downregulated (purple) miRNAs among those examined here. Stably expressed miRNAs are also shown (black). The ordinate axis indicates the relative amount of expression in a specific miRNA locus, and the horizontal axis indicates various types of isomiRs. Blue lines indicate isomiRs from HepG2 cells, and the red line indicates isomiRs from L02 cells. Some miRNAs with 3′ additional nucleotides are also highlighted in the horizontal axis. Stably expressed miRNAs show similar patterns of expression, and deregulated miRNAs show various patterns of expression in those cells. The figure only lists isomiRs (>50 for deregulated miRNAs, >100 for stably expressed miRNAs) for which normalized data was available.

IsomiR repertoires and expression profiles in the HepG2 and L02 cells were also analyzed. Various isomiR repertoires and expression patterns were detected in different miRNA loci (Figure 2). As found by Guo et al., several dominant isomiRs (always 1–3) were yielded per miRNA locus due to alternative and imprecise cleavage of Drosha and Dicer [34, 40]. Deregulated miRNAs might show abnormal isomiR expression profiles in tumor cells, such as miR-194-5p (upregulated) and miR-24-3p (downregulated) (Figure 2). These findings indicated inconsistent dominant isomiR sequences, even though they were always 5′ isomiRs with the same 5′ ends and seed sequences. This phenomenon was detected primarily in deregulated miRNAs. Generally, those stably expressed species had similar expression profiles between tumor and normal cells. This was true of miR-26a-5p and miR-21-5p (Figure 2). Of the dominant isomiRs, only miR-15a-5p was involved in 3′ addition (Figure 2). Although 3′ addition was quite widespread, especially for adenine and uracil, the presence of type of isomiRs with 3′ additions suggested considerable divergence between miRNAs. For example, miR-103a-3p was not detected any modified isomiRs even though 10 isomiRs were obtained, while three isomiRs with 3′ additions were found in miR-194-5p (Figure 2). At the miRNA locus, these modified isomiRs always possessed lower enrichment levels than dominant isomiR sequences, although they might still show high levels of expression.

Functional enrichment analysis was performed based on targets that had been found to be regulated by at least 2 abnormal miRNAs. The results suggested that these miRNAs play important roles in essential biological processes, including the cell cycle, Wnt, and the MAPK signaling pathway (Table 2). They also contribute to various human diseases, such as chronic myeloid leukemia, prostate cancer, and bladder cancer. According to identified mRNA profiles by using microarray technology, these target mRNAs might be stably expressed, upregulated, or downregulated in tumor cells.

### 3.2. Aberrantly Expressed mRNA and lncRNA Profiles

Dominant (>10 of normalized data) and significantly differentially expressed (fold change (log 2) >4.0 or <−4.0) mRNAs and lncRNAs (the top deregulated species could be found in Table 3) were collected. Significant divergence was detected between upregulated and downregulated RNA species. 83.98% of deregulated mRNAs and 90.93% deregulated lncRNAs were upregulated in tumor cells. However, the analysis of differentially expressed profiles suggested that 62.06% of mRNAs and 66.49% of lncRNAs were downregulated. Locational distributions of mRNA and lncRNA were analyzed. Consistent distribution patterns were detected, and no bias was found between deregulated mRNA and lncRNA species (Figures S2A, S2B, and S2C). However, inconsistent distributions were detected between the top 100 dominant and deregulated mRNAs and lncRNAs (Figures S2D and S2E). These abnormal species were prone to locate on chromosomes 6 and 12 (mRNA) and chromosomes 4 and 5 (lncRNA).

**Figure 3:**
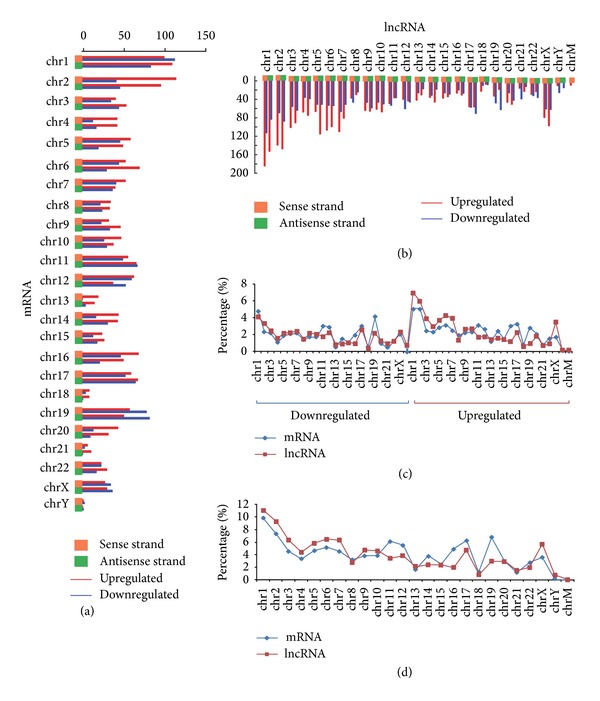
Distribution of aberrantly expressed mRNA and lncRNA profiles. Location distributions of aberrantly expressed (a) mRNA and (b) lncRNA profiles on human chromosomes. The number of deregulated species, including detailed up- and downregulated mRNAs and lncRNAs, is given with respect to sense and antisense strands, respectively. “chr”: chromosome; “chrM”: mitochondrial chromosome. (c) Distribution of upregulated and downregulated species. (d) Distribution of total deregulated mRNAs and lncRNAs.

Significantly deregulated mRNAs and lncRNAs were found to be prone to be located on chromosomes 1 and 2, especially upregulated species (Figures 3(a), 3(b), and 3(c)). Upregulation was found to be more common than downregulation (Figure 3). Generally, no significant distribution bias was found between sense and antisense strands (Figures 3(a) and 3(b)). Coding and noncoding RNAs indicated similar ratios of downregulated species, while they showed diversity of upregulated species (Figure 3(c)). Inconsistent locational distribution patterns were detected based on the total number of down- and upregulated mRNA and lncRNA species (Figure 3(d)).

**Figure 4:**
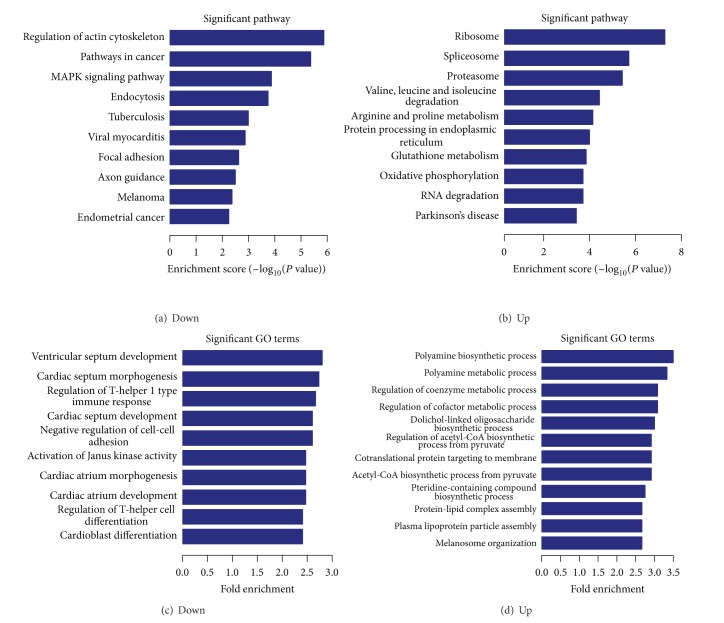
Analysis of significant pathways and GO terms regarding biological processes associated with abnormally expressed mRNA profiles in HepG2 cells. The *P* value denotes the significance of the pathway correlated to the conditions and GO term (the recommend *P* value cutoff is 0.05).

The pathway and GO analysis of abnormal mRNA expression profiles showed various results (Figure 4 and Figure S3). Downregulated mRNAs were prone to be found in pathways of regulation of actin cytoskeleton and pathways in cancer, and upregulated mRNAs contributed to the biological processes of ribosomes and spliceosomes (Figure 4). Dominant abnormal mRNAs were collected for functional enrichment analysis. Some of them had important roles in diverse essential biological processes through involvement in the pathways, including purine metabolism, ribosomes, the cell cycle, glycolysis, and gluconeogenesis (Table S1). They also contributed to occurrence and development of some human diseases, such as Parkinson's disease and small-cell lung cancer.

### 3.3. ncRNA-mRNA Data Integration and Interactive Regulators in Tumorigenesis

According to functional enrichment analysis of abnormal miRNAs and mRNAs, the common pathways could be obtained using different genes (Table S2). This was mainly attributable to the selected threshold values of analyzed miRNA and mRNA species. Not all dominant deregulated miRNAs and mRNAs had direct relationships.

**Figure 5:**
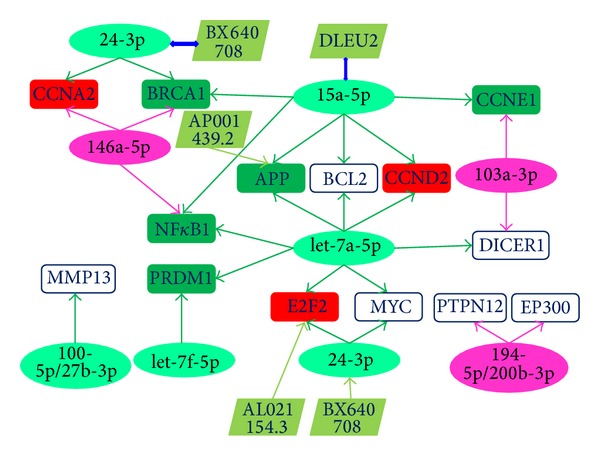
Interaction between deregulated ncRNA-mRNA in HepG2 cells. Each ellipse indicates up- (purple) or downregulated (light green) miRNA. Each square indicates up- (red) or downregulated (green) mRNA (stably expressed mRNAs are in white), and each tilted rectangle indicates downregulated lncRNAs (pale green). These miRNAs are shown in Table 1. A regulatory network was constructed using experimentally validated target mRNAs (each mRNA is regulated by at least 2 deregulated miRNAs). Stably expressed mRNAs are shown in white squares (*BCL2*,* MMP13*, and* PTPN12*). mRNAs not detected in HepG2 and L02 cells are also shown in white squares (*DICER1*,* EP300*, and* MYC*).

An analysis of miRNA-mRNA interactions showed a complex regulatory network (Figure 5). mRNAs that were regulated by at least 2 abnormally expressed miRNAs were collected. Generally, they were prone to form closed networks with close regulatory relationships. Some miRNAs, such as let-7a-5p and miR-15a-5p, were located in the central positions with multiple target mRNAs. Although small regulatory molecules were downregulated or upregulated, their target mRNAs might show consistent or inconsistent dysregulation patterns (Figure 5). Locational relationships indicated that related lncRNAs were also constructed in the regulatory network. Some miRNAs, such as miR-24-3p (mir-24-2 gene is located in BX640708), always showed consistent deregulation patterns with their host lncRNAs (Figure 5). Several mRNAs were also found to be related to nearby lncRNAs. mRNA and associated lncRNA might be located on the same strand or have a sense/antisense relationship within a specific genomic region. mRNA-lncRNA might show consistent (APP & AP001439.2) or inconsistent (E2F2 & AL021154.3) deregulation patterns (Figure 5).

**Figure 6:**
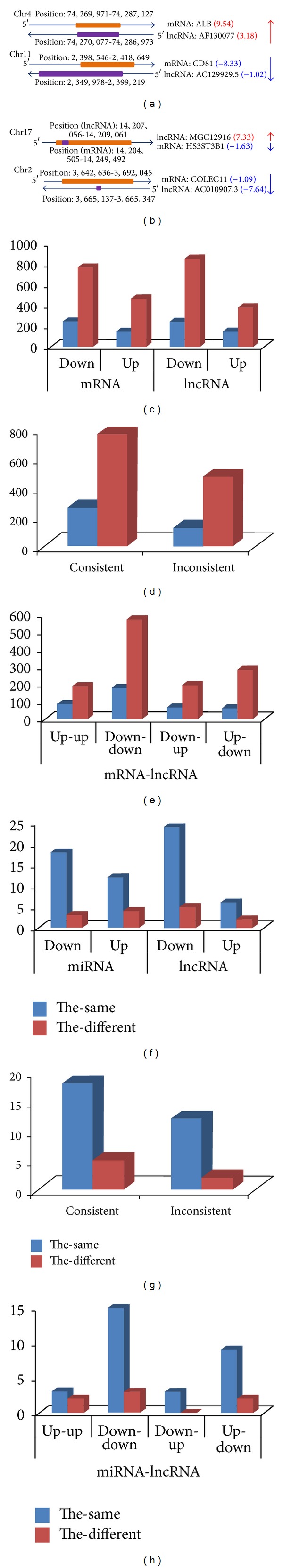
Integrative expression analysis of mRNA-lncRNA and miRNA-lncRNA based on their locations. ((a)-(b)) Schematic representation of expression of mRNA-lncRNA (Table 3). Some of these mRNA genes and lncRNA genes are always located on sense/antisense strands in specific genomic regions. Others are located in the same genomic region but are of different lengths. Some show the same deregulation patterns with different fold changes (log 2) (upregulation: red, downregulation: blue), and others show different deregulation patterns. ((c)–(e)) mRNA-lncRNA integrative analysis based on genomic location. The ordinate axis indicates the number of deregulated mRNAs or lncRNAs. The term “the-same” indicates that mRNA and lncRNA are located on the same strand. The term “the-different” indicates that mRNA and lncRNA are located on sense/antisense strands. ((f)–(h)) miRNA-lncRNA integrative analysis based on genomic location. The ordinate axis indicates the number of deregulated miRNAs or lncRNAs. The term “the-same” indicates that miRNA and lncRNA are located on the same strand. The term “the-different” indicates that miRNA and lncRNA are located on sense/antisense strands.

To identify the overall patterns of deregulation between mRNAs/miRNAs and lncRNAs, a comprehensive survey of their potential relationships was performed incorporating information regarding mRNAs, miRNAs, and lncRNAs. Most mRNA-lncRNA pairs had sense/antisense relationships, and miRNA-lncRNA pairs were prone to be located on the same strands (Table S3). Generally, these mRNA/miRNA-lncRNA pairs could completely or partially overlap (from the same strands) or show reverse complementarily binding (from sense/antisense strands). The mRNA and lncRNA could show the same or different deregulation patterns, but they were usually the same (Figure 6). Some pairs were up- or downregulated, and their fold change values differed (Figures 6(a) and 6(b)). These RNA molecules, both coding RNAs, which are functional molecules, and noncoding RNAs, which are regulatory molecules, were prone to be downregulated in tumor cells.

## 4. Discussion

Although the recorded values of differences in expression, as defined as the abundance of isomiRs, expression levels of isomiRs may have been influenced by higher sensitivity of next-generation sequencing technology, the diversity of those expression is mainly attributable to differences in the isomiR profiles and expression patterns in normal and tumor cells (Figure S4). Indeed, the most dominant isomiRs are not always canonical miRNA sequences. The wide range of these inconsistent sequences was found to contribute to various isomiR repertoires, leading to differences in expression between the most abundant isomiR and other isomiRs. Herein, 3′ addition is detected in many places, but it is not always present in the dominant sequences, though it may show high level of expression (Figure 2 and Figure S4). Stably expressed miRNAs always show similar isomiR patterns, and deregulated miRNA species are prone to show different isomiR repertoires (Figure 2) [34]. Generally, isomiR profiles remain stable in different tissues [31, 33, 34]. Deviant isomiR expression profiles should not be considered random events. These results strongly suggest that the isomiR repertoires and their patterns of expression might contribute to tumorigenesis through playing biological roles [34]. Collectively, the detailed isomiR repertoires might serve as markers and provide information regarding the regulatory mechanisms of small noncoding active molecules.

miRNAs are small negative regulatory molecules. They can suppress gene expression via mRNA degradation or repression of translation [4, 6]. However, integrative analysis shows both consistent and inconsistent deregulation patterns, indicating complex regulatory networks containing both noncoding RNAs and mRNAs (Figure 5). mRNAs are always regulated by multiple miRNAs, and vice versa. The dynamic expression patterns between miRNAs and mRNAs are more complex than had been believed. Even though multiple target mRNAs can be detected, miRNA may regulate specific mRNAs at specific times and at specific sites. Competitive interactions between miRNA and mRNA may be dynamic and involve complex regulatory mechanisms in a specific microenvironment. Dominant selection may exist in miRNA and mRNA. Selective, dynamic, flexible interactions may produce robust coding-noncoding RNA regulatory networks, especially networks involving lncRNAs. The robust regulatory patterns contribute to normal biological processes. Abnormal regulatory networks may produce aberrant pathways and even disease. Specifically, let-7-5p has been experimentally validated as crucial regulatory molecule in hepatocellular carcinoma. They can negative regulate Bcl-xL expression and strengthen sorafenib-induced apoptosis [41], and can contribute to protecting human hepatocytes from oxidant injury through regulating Bach1 [42]. Common pathways can be produced through enrichment analysis of miRNAs and mRNAs (Table S2). Significantly up- and downregulated mRNAs show various pathways and GO terms (Figure 4 and Figure S3). The large number and variety of biological roles and their positions in pathways and networks indicate that they may contribute to tumorigenesis. Assessing the actual interaction networks can be difficult* in vivo* because of dynamic expression, although high-throughput techniques can be used to track and construct whole-expression profiles.

Both dominant and deregulated miRNAs are prone to be located on specific chromosomes (Figure S1D). The bias might implicate active transcription of specific regions or chromosomes. However, abnormal species do not show distribution biases (Figure 3 and Figure S2). Among dominant aberrantly expressed miRNA, downregulation is quite common. Among mRNAs and lncRNAs, upregulation is more common. All of these findings suggest consistent or coexpression patterns shared by mRNAs and lncRNAs. These are mainly derived from original transcription from genomic DNA sequences. miRNAs and mRNAs/lncRNAs tended to show opposite deregulation patterns. Evidence suggests that miRNA can regulate lncRNA through methylation. For example, miR-29 can regulate the long noncoding gene* MEG3* in hepatocellular cancer through promoter hypermethylation [43]. Moreover, some miRNAs are encoded by exons of long noncoding transcripts [44, 45]. These miRNAs and their host gene lncRNAs may be cotranscribed and coregulated. These would include miR-31 and its host gene, lncRNA* LOC554202*, which, in triple-negative breast cancer, are regulated through promoter hypermethylation [46].

Noncoding RNA molecules, especially miRNAs and lncRNAs, are very prevalent regulatory molecules. They have been shown to play versatile roles in many biological processes. These usually involve transcriptional regulation and modulation of protein function [23]. In the present study, the nearness or separation of ncRNAs and mRNAs on the chromosome is used to perform a comprehensive analysis. Results show that mRNA-lncRNA pairs always have consistent or inconsistent deregulation patterns (Figures 5 and 6 and Table S3). Although some pairs have sense/antisense relationships, the same trends can be detected even at differences in fold change of far greater magnitude (log 2) (Figures 6(a) and 6(b)). The various fold change indicates different degrees of up- or downregulation between mRNAs and lncRNAs. This information might be used to determine the method of regulation. The two members of each mRNA-lncRNA pair can overlap completely or partially (on the same strand). Some of them can also form duplexes through reverse complementary binding (from the sense/antisense strands), which may facilitate interactions between different RNA molecules. The pronounced divergence with respect to the degree of deregulation might be attributable to different regulatory methods, although other complex mechanisms may also be involved. The mRNA and lncRNA from the same strands sometimes show opposite deregulation trends (Figure 6(b)), although the fact that they are cotranscribed from the same genomic DNA sequence with similar original expression levels. Complex negative regulatory networks, especially those involved in noncoding miRNAs and lncRNAs, contribute to the diversity of final relative expression levels* in vivo*. Abnormal regulation in the coding-noncoding RNA network may be pivotal to tumorigenesis.

miRNA-lncRNA pairs with locational relationships are also surveyed. The miRNA and lncRNA in these pairs are more prone to be located on the same strand with complete overlap than paired mRNA and lncRNA molecules are (Figure 6 and Table S3). They may show either consistent or inconsistent deregulation patterns, but consistent patterns are more common (Figure 6). The different levels of final enrichment may be attributable to degradation and regulatory mechanisms. Diversity of abnormal half-life for specific RNA molecules may cause the development of diseases. However, although molecules may have different regulatory relationships, a robust regulatory network can be detected, especially due to multiple targets of each molecule. Alternative regulatory pathways, particularly flexible candidate regulatory pathways and functional pathways, indicate that the coding-noncoding RNA regulatory network is more complex than had been believed, especially in different space and time. The possible flexible relationships between molecules in various places and at various times are crucial to determining the mechanism underlying tumorigenesis.

## Supplementary Material

Table S1 and Table S2 presented functional enrichment analysis of dominant abnormal mRNAs and mRNAs; Table S3 showed location distributions based on the integrative analysis of miRNAs, mRNAs and lncRNAs; Figure S1 and Figure S2 showed location distribution patterns of miRNAs, mRNAs and lncRNAs; Figure S3 presented graphical representation of GO-term enrichment analysis of abnormal mRNA expression profiles; Figure S4 presented the divergence of isomiR repertoires between HepG2 and L02 cells.

## Figures and Tables

**Table 1:** Differentially expressed abundant miRNA species as indicated by the most abundant isomiR and all isomiRs.

miRNA	Chr	Consistent or inconsistent	Fold change (the most)	Fold change (all isomiRs)	Up/down
let-7a-5p	9, 11, 22	Yes	−2.35	−3.24	Down
let-7f-5p	9, X	Yes	−2.54	−2.97	Down
miR-103a-3p	5, 20	Yes	2.33	2.05	Up
miR-146a-5p	5	Yes	7.28	5.52	Up
miR-15a-5p	13	No	−4.85	−3.87	Down
miR-194-5p	1, 11	No	7.26	4.56	Up
miR-200b-3p	1	No	8.56	5.45	Up
miR-23a-3p	19	No	−7.19	−5.02	Down
miR-24-3p	9, 19	No	−4.24	−2.14	Down
miR-27a-3p	19	Yes	−6.61	−6.35	Down
miR-27b-3p	9	Yes	−1.82	−2.61	Down
miR-100-5p	11	Yes	−7.27	−4.88	Down
miR-425-5p	3	No	2.98	2.00	Up

These miRNAs are abundantly expressed in HepG2 and L02 cells. They are the top downregulated and upregulated miRNAs in cancer cells (fold change (log⁡2) >2.0 or <−2.0). Chr indicates the genomic locations of the miRNA genes (pre-miRNAs), including multicopy pre-miRNAs. let-7a-5p is located on chr9 (let-7a-1), 11 (let-7a-2), and 22 (let-7a-3). The term “consistent” indicates that the sequence of the most abundant isomiR is the same as that of the reference miRNA sequence in the miRBase database. The term “most” indicates the most abundant isomiR from a given locus. The term “all isomiRs” indicates total number of isomiRs from a given locus.

**Table 2:** Pathway enrichment analysis of experimentally validated mRNA targets of dominant deregulated miRNAs.

Pathway	Number	*P* value	Target genes
Cell cycle	18	3.01*E* − 30	*ATM; * ***CCNA2***; *CCND1; * ***CCND2***; *CCNE1; CDC25A; CDK6; CDKN1A; CDKN1B; CDKN2A; E2F1; * ***E2F2***; *E2F3; EP300; RB1; * ***RBL2***; *TP53; WEE1*

Chronic myeloid leukemia	15	2.74*E* − 27	*ACVR1C; AKT1; CCND1; CDK6; CDKN1A; CDKN1B; CDKN2A; E2F1; * ***E2F2***; *E2F3; MYC; NFKB1; * ***NRAS***; *RB1; TP53 *

Prostate cancer	15	3.74*E* − 26	*AKT1; BCL2; CCND1; CCNE1; CDKN1A; CDKN1B; E2F1; * ***E2F2***; *E2F3; EP300; IGF1R; NFKB1; * ***NRAS***; *RB1; TP53 *

Pancreatic cancer	14	3.03*E* − 25	*ACVR1C; AKT1; CCND1; * ***CDC42***; *CDK6; CDKN2A; E2F1; * ***E2F2***; *E2F3; NFKB1; RAC1; RB1; TP53; VEGFA *

Bladder cancer	13	1.47*E* − 26	*CCND1; CDKN1A; CDKN2A; E2F1; * ***E2F2***; *E2F3; FGFR3; MYC; * ***NRAS***; *RB1; THBS1; TP53; VEGFA *

Melanoma	13	3.21*E* − 23	*AKT1; CCND1; CDK6; CDKN1A; CDKN2A; E2F1; * ***E2F2***; *E2F3; IGF1R; * ***MET***; ***NRAS***; *RB1; TP53 *

Melanoma	13	3.21*E* − 23	*AKT1; CCND1; CDK6; CDKN1A; CDKN2A; E2F1; * ***E2F2***; *E2F3; IGF1R; * ***MET***; ***NRAS***; *RB1; TP53 *

Small-cell lung cancer	13	4.71*E* − 22	*AKT1; BCL2; CCND1; CCNE1; CDK6; CDKN1B; E2F1; * ***E2F2***; *E2F3; MYC; NFKB1; RB1; TP53 *

Focal adhesion	13	5.65*E* − 17	*AKT1; BCL2; CCND1; * ***CCND2***; ***CDC42***; *PAK3; IGF1R; * ***MET***; *RAC1; RHOA; ROCK1; THBS1; VEGFA *

Glioma	12	1.49*E* − 21	*AKT1; CCND1; CDK6; CDKN1A; CDKN2A; E2F1; * ***E2F2***; *E2F3; IGF1R; * ***NRAS***; *RB1; TP53 *

Nonsmall cell lung cancer	10	3.77*E* − 18	*AKT1; CCND1; CDK6; CDKN2A; E2F1; * ***E2F2***; *E2F3; * ***NRAS***; *RB1; TP53 *

Renal cell carcinoma	10	7.11*E* − 17	*AKT1; * ***CDC42***; *PAK3; EP300; ETS1; * ***HIF1A***; ***MET***; ***NRAS***; *RAC1; VEGFA *

Axon guidance	10	3.77*E* − 14	***CDC42***; *PAK3; CXCL12; CXCR4; * ***MET***; *NFAT5; * ***NRAS***; *RAC1; RHOA; ROCK1 *

p53 signaling pathway	9	1.83*E* − 14	*ATM; CCND1; * ***CCND2***; *CCNE1; CDK6; CDKN1A; CDKN2A; THBS1; TP53 *

Colorectal cancer	9	1.02*E* − 13	*ACVR1C; AKT1; BCL2; CCND1; IGF1R; * ***MET***; *MYC; RAC1; TP53 *

Wnt signaling pathway	9	1.88*E* − 11	*CCND1; * ***CCND2***; *EP300; MYC; NFAT5; RAC1; RHOA; ROCK1; TP53 *

MAPK signaling pathway	9	2.68*E* − 09	*ACVR1C; AKT1; * ***CDC42***; *FGFR3; MYC; NFKB1; * ***NRAS***; *RAC1; TP53 *

Adherens junction	8	4.04*E* − 12	*ACVR1C; * ***CDC42***; *EP300; IGF1R; * ***MET***; *RAC1; RHOA; WASF3 *

These target mRNAs are found to be regulated by at least 2 abnormal miRNAs each. Bold type indicates upregulation. Underlining indicates downregulation. Other fonts indicate stable expression or undetectable levels.

**Table 3:** Differentially expressed abundant mRNA and lncRNA species.

mRNA/lncRNA	Gene symbol	Chr (±)	HepG2 (Nor)	L02 (Nor)	Fold change	Up/down
mRNA	RBP4	chr10 (−)	16.41	4.90	11.51	Up
mRNA	APOA1	chr11 (−)	15.07	5.44	9.64	Up
mRNA	ALB	chr4 (+)	14.19	4.65	9.54	Up
mRNA	ID2	chr2 (+)	13.52	4.16	9.35	Up
mRNA	TFPI	chr2 (−)	13.26	4.29	8.97	Up
mRNA	SERPINA3	chr14 (+)	14.15	5.42	8.73	Up
mRNA	SRGN	chr10 (+)	5.26	13.59	−8.33	Down
mRNA	CD81	chr11 (+)	4.66	12.99	−8.33	Down
mRNA	FOLR1	chr11 (+)	5.00	14.50	−9.50	Down
mRNA	NNMT	chr11 (+)	3.74	13.28	−9.54	Down
mRNA	C11orf86	chr11 (+)	4.26	15.16	−10.90	Down
mRNA	BASP1	chr5 (+)	5.53	17.06	−11.53	Down
lncRNA	RP11-113C12.1	chr12 (−)	12.71	5.43	7.28	Up
lncRNA	D28359	chr13 (+)	13.95	6.63	7.31	Up
lncRNA	MGC12916	chr17 (+)	11.25	3.91	7.33	Up
lncRNA	ABCC6P1	chr16 (+)	12.28	4.89	7.39	Up
lncRNA	lincRNA-HEY1	chr8 (−)	12.12	4.70	7.42	Up
lncRNA	HSPEP1	chr20 (−)	13.22	5.60	7.61	Up
lncRNA	TMSL6	chr20 (−)	8.25	16.08	−7.83	Down
lncRNA	RP11-163G10.3	chr1 (−)	8.18	15.92	−7.74	Down
lncRNA	AC010907.3	chr2 (−)	7.44	15.07	−7.64	Down
lncRNA	BC106081	chr8 (−)	3.70	10.81	−7.11	Down
lncRNA	nc-HOXA11-86	chr7 (+)	3.83	10.65	−6.82	Down
lncRNA	AK054970	chr13 (+)	6.01	12.64	−6.62	Down

The table only lists the top 6 up- and downregulated mRNAs and lncRNAs based on the fold change values (log⁡2). These mRNAs and lncRNAs are dominantly expressed. “Chr (±)” indicates genomic location on sense or antisense strands of human chromosomes. “Nor” indicates the normalized data.

## Abbreviations

ncRNA: Noncoding RNAs
miRNA: MicroRNA
isomiR: miRNA variant
lncRNA: Long noncoding RNA
mRNA: Messenger RNA
pre-miRNA: Precursor miRNA
RPM: Reads per million.
