# Supplementary material for: An Integrated Analysis of miRNA, lncRNA, and mRNA Expression Profiles
Source: Biomed Res Int. 2014 Jun 18;2014:345605. doi: 10.1155/2014/345605 (PMC4086520; doi:10.1155/2014/345605)
Supplement: Supplementary file 1 — Table S1 and Table S2 presented functional enrichment analysis of dominant abnormal mRNAs and mRNAs; Table S3 showed location distributions based on the integrative analysis of miRNAs, mRNAs and lncRNAs; Figure S1 and Figure S2 showed location distribution patterns of miRNAs, mRNAs and lncRNAs; Figure S3 presented graphical representation of GO-term enrichment analysis of abnormal mRNA expression profiles; Figure S4 presented the divergence of isomiR repertoires between HepG2 and L02 cells. [file 345605.f1.doc]

**An Integrated Analysis of miRNA, lncRNA and mRNA Expression Profiles**

Li Guo^*^, Yang Zhao, Sheng Yang, Hui Zhang, Feng Chen*

Department of Epidemiology and Biostatistics, School of Public Health, Nanjing Medical University, 211166, China

* To whom correspondence should be addressed.

**Tel:** +86-25-86868436

**Fax:** +86-25-86868436

E-mail addresses:

LG: [gl8008@163.com](mailto:gl8008@163.com), [lguo@njmu.edu.cn](mailto:lguo@njmu.edu.cn)

FC: [fengchen@njmu.edu.cn](mailto:fengchen@njmu.edu.cn)

**Supplemental materials**

**Table S1.** Pathway enrichment analysis of dominant abnormal mRNAs.

| **Pathway/network** | **No.** | **P-value** | **Target Genes** |
| --- | --- | --- | --- |
| Purine metabolism | 8 | 2.50E-06 | *POLR2D;POLR1D;NME4;AK3L1;PPAT;PRPS1;PRPS1L1;POLR1C;POLR1D;POLR2D* |
| Ribosome | 7 | 1.84E-06 | *RPL7;RPS10;RPL4;RPL27;FAU;RPL35A;RPSA* |
| Glycolysis / Gluconeogenesis | 6 | 1.64E-06 | *PGAM4;BPGM;ADH6;LDHA;HK2;ADH5* |
| Cell cycle | 6 | 5.88E-05 | *YWHAG;YWHAQ;RBX1;PTTG1;YWHAH;BUB3* |
| MAPK signaling pathway | 6 | 4.69E-03 | *DUSP9;HSPA8;DDIT3;STK4;RPS6KA3;MAP4K2* |
| Pyrimidine metabolism | 5 | 1.83E-04 | *POLR2D;POLR1D;NME4;AK3;POLR1C;POLR1D;POLR2D* |
| Parkinson's disease | 5 | 1.04E-03 | *SLC25A5;COX7B;NDUFB6;VDAC2;NDUFS4* |
| Oxidative phosphorylation | 5 | 1.15E-03 | *COX7B;ATP6V1F;NDUFB6;PPA1;NDUFS4* |
| Wnt signaling pathway | 5 | 1.70E-03 | *FZD4;RBX1;DVL1;LRP5;FRAT2* |
| Regulation of actin cytoskeleton | 5 | 7.85E-03 | *ARPC1B;FN1;ARPC1A;LIMK2;F2* |
| Pathogenic Escherichia coli infection - EHEC | 4 | 2.60E-04 | *YWHAQ;TUBB3;TUBB2A;TUBB6* |
| Pathogenic Escherichia coli infection - EPEC | 4 | 2.60E-04 | *YWHAQ;TUBB3;TUBB2A;TUBB6* |
| Complement and coagulation cascades | 4 | 6.19E-04 | *F3;TFPI;SERPINA1;F2* |
| Antigen processing and presentation | 4 | 1.60E-03 | *PSME1;HSPA8;HLA-B;PDIA3* |
| Ubiquitin mediated proteolysis | 4 | 7.88E-03 | *RBX1;TCEB1;TRIM37;WWP1* |

A total of 400 dominant aberrantly expressed mRNAs are performed the functional enrichment analysis. 105 of them are enriched 99 pathways/networks. Here, we only list some that are involved at least 4 mRNAs.

**Table S2.** Common pathway of dominant abnormal mRNAs and miRNAs.

| **Pathway** | **Genes (mRNAs)** | **Target Genes (miRNAs)** |
| --- | --- | --- |
| Adherens junction | *PTPRF;PVRL2 (2)* | *ACVR1C;CDC42;EP300;IGF1R;MET;RAC1;RHOA;WASF3 (8)* |
| Apoptosis | *AIFM1;CFLAR (2)* | *AKT1;ATM;BCL2;NFKB1;TP53 (5)* |
| Axon guidance | *SEMA4C;LIMK2 (2)* | *CDC42;PAK3;CXCL12;CXCR4;MET;NFAT5;NRAS;RAC1;RHOA;ROCK1 (10)* |
| Cell cycle | *YWHAG;YWHAQ;RBX1;PTTG1;YWHAH;BUB3 (6)* | *ATM;****CCNA2****;CCND1;CCND2;CCNE1;CDC25A;CDK6;CDKN1A;CDKN1B;CDKN2A;E2F1;E2F2;E2F3;EP300;RB1;RBL2;TP53;WEE1 (18)* |
| Colorectal cancer | *FZD4;DVL1 (2)* | *ACVR1C;AKT1;BCL2;CCND1;IGF1R;MET;MYC;RAC1;TP53 (9)* |
| Cytokine-cytokine receptor interaction | *LTBR;EDA (2)* | *CXCL12;CXCR4;MET;VEGFA (4)* |
| Focal adhesion | *SPP1;FN1 (2)* | *AKT1;BCL2;CCND1;CCND2;CDC42;PAK3;IGF1R;MET;RAC1;RHOA;ROCK1;THBS1;VEGFA (13)* |
| Insulin signaling pathway | *PTPRF;INPP5D (2)* | *AKT1;NRAS (2)* |
| MAPK signaling pathway | *DUSP9;HSPA8;DDIT3;STK4;RPS6KA3;MAP4K2 (6)* | *ACVR1C;AKT1;CDC42;FGFR3;MYC;NFKB1;NRAS;RAC1;TP53 (9)* |
| Melanogenesis | *FZD4;DVL1 (2)* | *EP300;NRAS (2)* |
| Pathogenic Escherichia coli infection - EHEC | *YWHAQ;TUBB3;TUBB2A;TUBB6 (4)* | *CDC42;RHOA;ROCK1 (3)* |
| Pathogenic Escherichia coli infection - EPEC | *YWHAQ;TUBB3;TUBB2A;TUBB6 (4)* | *CDC42;RHOA;ROCK1 (3)* |
| PPAR signaling pathway | *APOA1;CPT1A (2)* | *PPARA;PPARG (2)* |
| Regulation of actin cytoskeleton | *ARPC1B;FN1;ARPC1A;LIMK2;F2 (5)* | *CDC42;PAK3;FGFR3;NRAS;RAC1;RHOA;ROCK1 (7)* |
| Renal cell carcinoma | *RBX1;TCEB1 (2)* | *AKT1;CDC42;PAK3;EP300;ETS1;HIF1A;MET;NRAS;RAC1;VEGFA (10)* |
| TGF-beta signaling pathway | *ID2;RBX1;SMAD7 (3)* | *ACVR1C;EP300;MYC;RBL2;RHOA;ROCK1;THBS1 (7)* |
| Wnt signaling pathway | *FZD4;RBX1;DVL1;LRP5;FRAT2 (5)* | *CCND1;CCND2;EP300;MYC;NFAT5;RAC1;RHOA;ROCK1;TP53 (9)* |

A total of 156 pathways/networks are obtained according to abnormal mRNAs (99) and miRNAs (57). 38 common pathways are identified with different genes. The table only lists that are involved at least 2 genes. The number of genes is also listed.

**Table S3.** Integrative analysis of miRNAs, mRNAs, and lncRNAs based on their location distributions.

| **RNA-RNA** | **The same stand** | **The different strand** | **Total** |
| --- | --- | --- | --- |
| mRNA-lncRNA | 392 | 1,239 | 1,631 |
| miRNA-lncRNA | 30 | 7 | 37 |

The same strand indicates that mRNA/miRNA-lncRNA genes are located on the same strand in a specific genomic region. Their sequences are complete or part overlapped. The different strand indicates that mRNA/miRNA-lncRNA genes are located on sense/antisense strands. They have sense/antisense relationships.

**
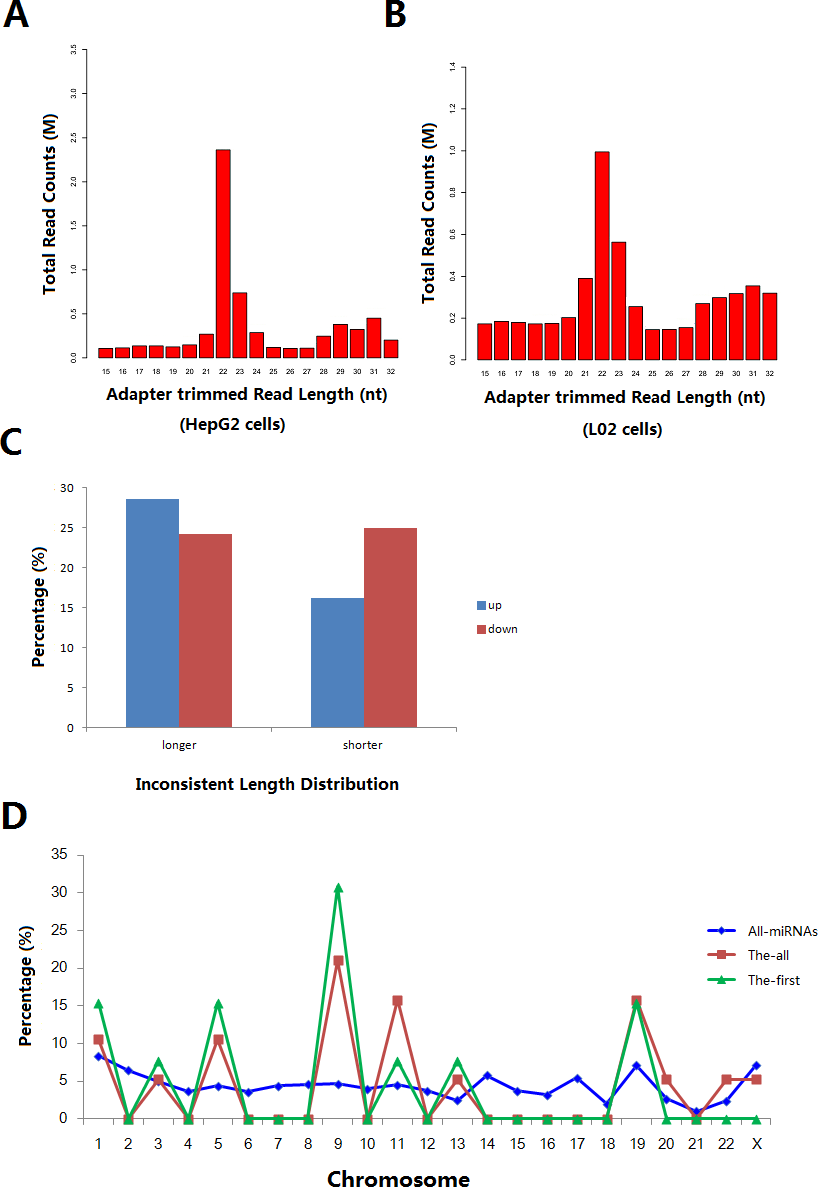
**

**Fig. S1.** Length and location distribution patterns of miRNAs.

**
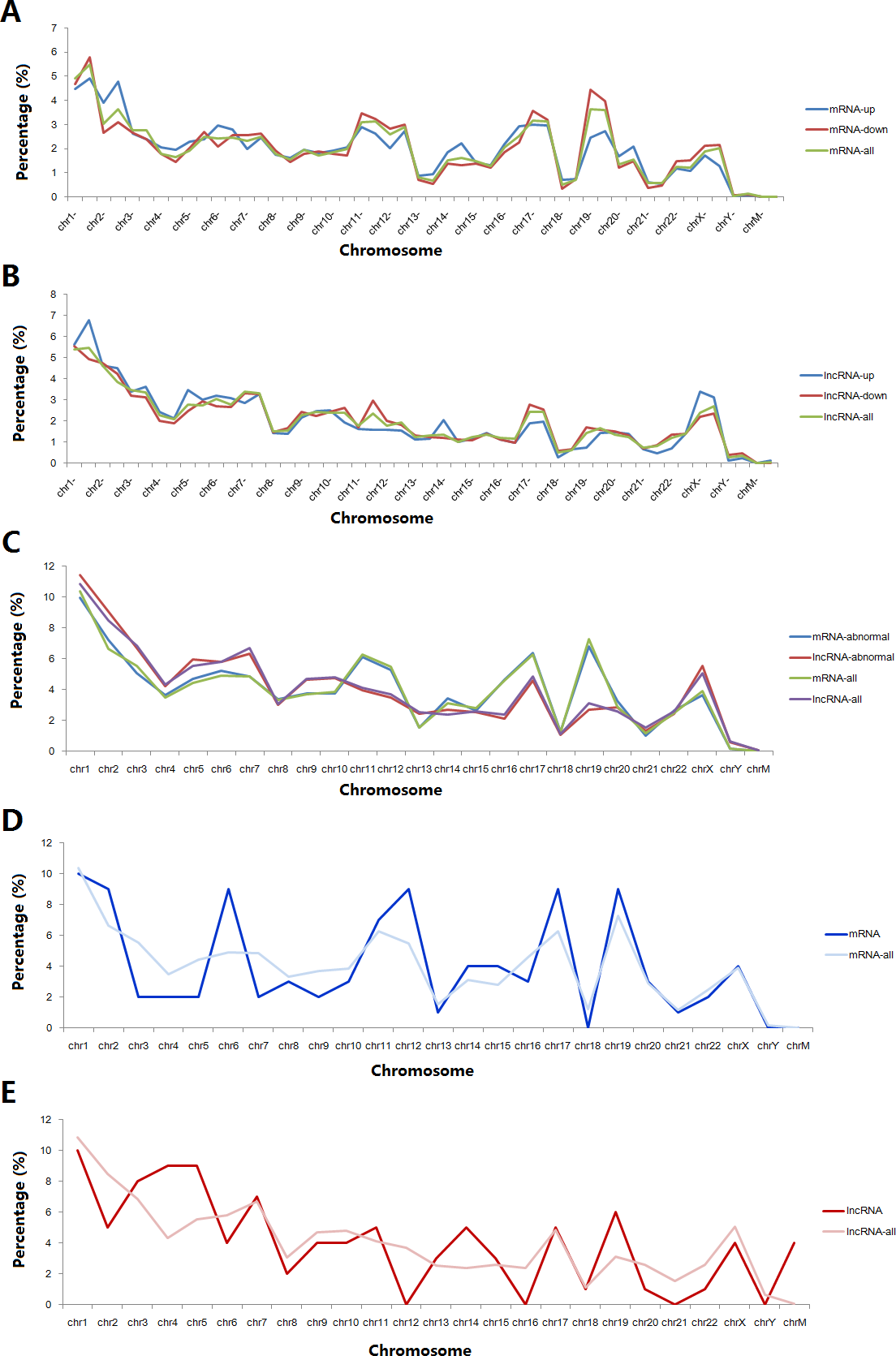
**

**Fig. S2.** Location distributions of abnormally and expression profiles of mRNAs and lncRNAs.

**
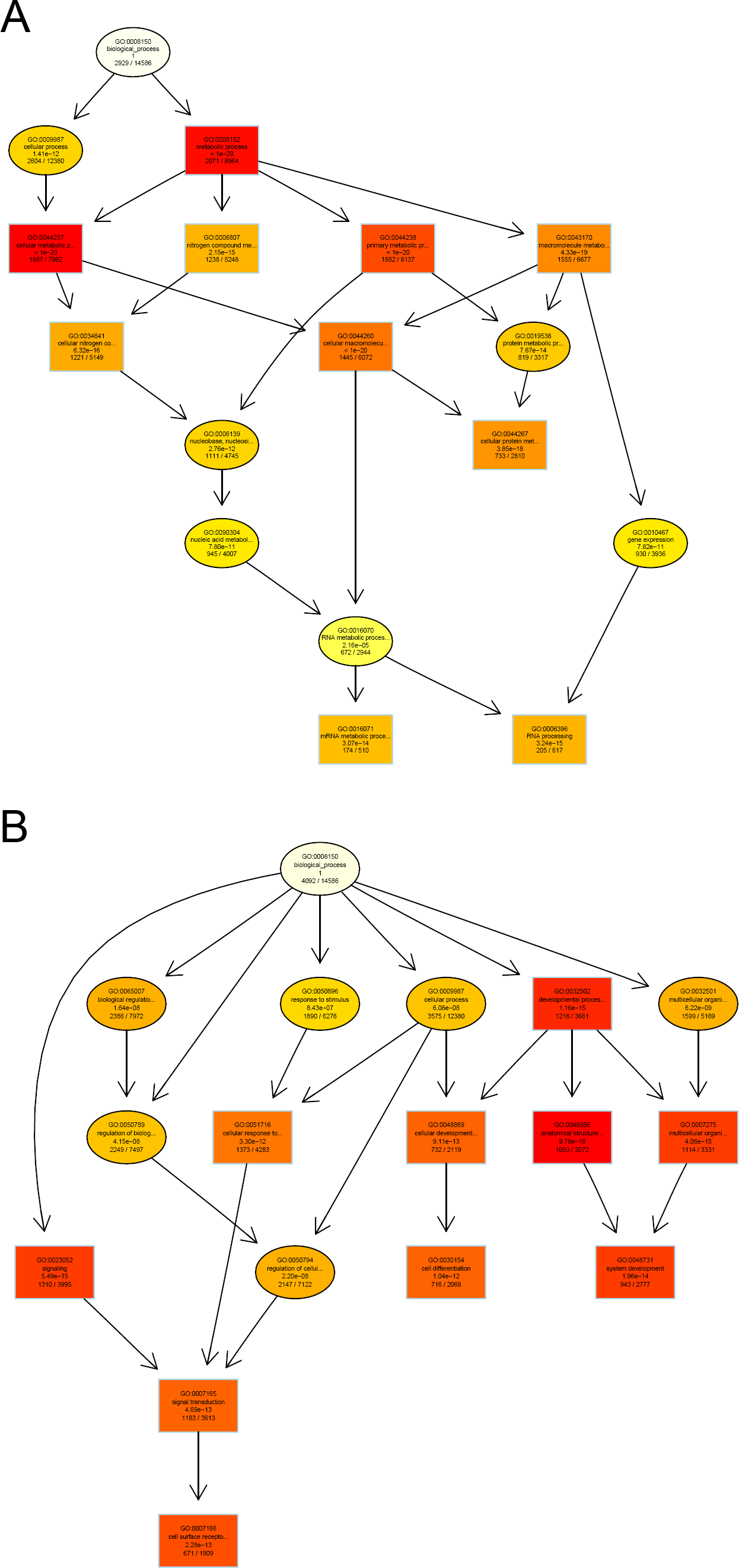
**

**Fig. S3.** Graphical representation of GO-term enrichment analysis of (A) up-regulated and (B) down-regulated mRNA profiles.

**
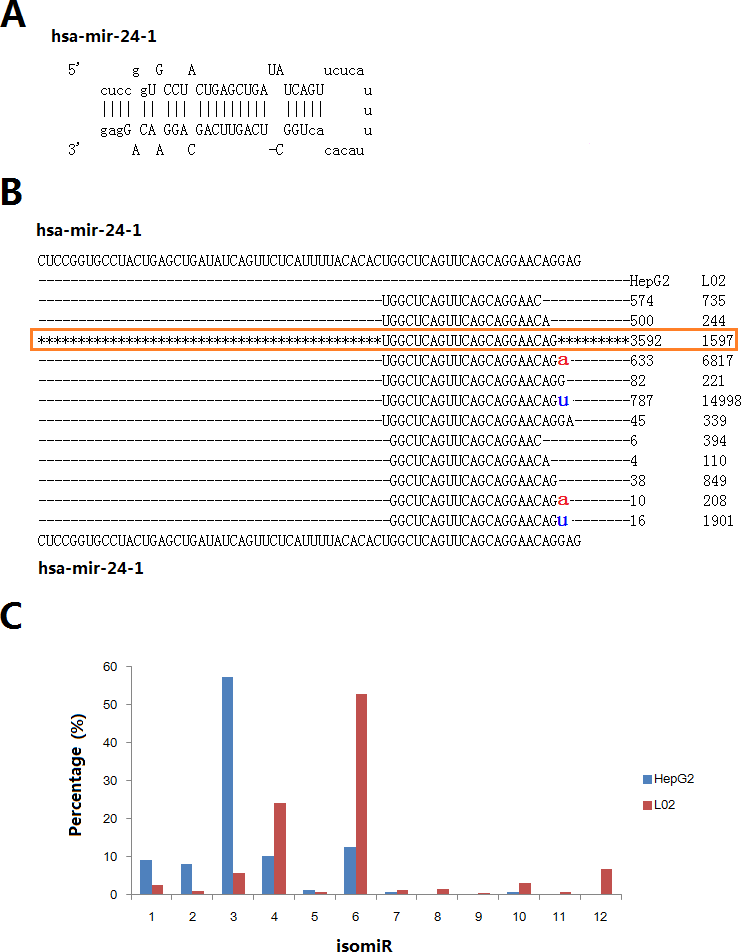
**

**Fig. S4.** Divergence of isomiR repertoires between HepG2 and L02 cells.
